# The Rostock International Parkinson's Disease (ROPAD) Study: Protocol and Initial Findings

**DOI:** 10.1002/mds.28416

**Published:** 2020-12-14

**Authors:** Volha Skrahina, Hanaa Gaber, Eva‐Juliane Vollstedt, Toni M. Förster, Tatiana Usnich, Filipa Curado, Norbert Brüggemann, Jefri Paul, Xenia Bogdanovic, Selen Zülbahar, Maria Olmedillas, Snezana Skobalj, Najim Ameziane, Peter Bauer, Ilona Csoti, Natalia Koleva‐Alazeh, Ulrike Grittner, Ana Westenberger, Meike Kasten, Christian Beetz, Christine Klein, Arndt Rolfs, the ROPAD Study Group

**Affiliations:** ^1^ CENTOGENE GmbH Rostock Germany; ^2^ Institute of Neurogenetics University of Lübeck Lübeck Germany; ^3^ Parkinson‐Center Gertrudisklinik Biskirchen Leun Germany

**Keywords:** Parkinson's disease, observational clinical study, genetic factors, *LRRK2*, *GBA*

## Abstract

**Background:**

Genetic stratification of Parkinson's disease (PD) patients facilitates gene‐tailored research studies and clinical trials. The objective of this study was to describe the design of and the initial data from the Rostock International Parkinson's Disease (ROPAD) study, an epidemiological observational study aiming to genetically characterize ~10,000 participants.

**Methods:**

Recruitment criteria included (1) clinical diagnosis of PD, (2) relative of participant with a reportable *LRRK2* variant, or (3) North African Berber or Ashkenazi Jew. DNA analysis involved up to 3 successive steps: (1) variant (*LRRK2*) and gene (*GBA*) screening, (2) panel sequencing of 68 PD‐linked genes, and (3) genome sequencing.

**Results:**

Initial data based on the first 1360 participants indicated that the ROPAD enrollment strategy revealed a genetic diagnostic yield of ~14% among a PD cohort from tertiary referral centers.

**Conclusions:**

The ROPAD screening protocol is feasible for high‐throughput genetic characterization of PD participants and subsequent prioritization for gene‐focused research efforts and clinical trials. © 2020 The Authors. *Movement Disorders* published by Wiley Periodicals LLC on behalf of International Parkinson and Movement Disorder Society.

Parkinson's disease (PD) is the most prevalent neurodegenerative movement disorder.^1^ Although the disease etiology remains elusive in the majority of cases, a multitude of monogenic forms for PD were described over the last 2 decades.^2^ Meanwhile, variants in *LRRK2* and *GBA* are recognized as being the most frequent cause^3^, ^4^ and risk factor,^5^ respectively. The frequencies of these variants are particularly high in specific ethnicities (eg, Ashkenazi Jews and North African Berbers). Although numerous additional, less frequent forms of monogenic PD have been identified,^6^ their overall rarity hampers the definition of the corresponding prevalence and mutational spectra by smaller‐scale studies. Moreover, the well‐documented heritability of the disease suggests that comprehensive genetic screening efforts will identify further PD‐linked genes.^7^ Finally, a multitude of other genetic conditions may initially manifest as PD.^8^, ^9^ Despite the evident role of genetic factors, genetic testing of clinically diagnosed PD patients is not part of the standard‐of‐care testing,^10^ impeding the identification of candidate participants for gene‐tailored research studies and clinical trials.

The present article introduces the Rostock International Parkinson's Disease (ROPAD) study, a recently initiated observational clinical study that enrolls participants for comprehensive genetic screening. It describes the general concept of the study, outlines its specific approaches, and presents preliminary data from the first 8 months of recruitment and analysis.

## Patients and Methods

### General Setting

The ROPAD study is an observational clinical study in a multicenter international setting. Its major goals are to (1) better understand the overall contribution of genetics to PD, (2) inform the downstream Lübeck International Parkinson's Disease (LIPAD) study (see below) and thereby support the characterization of specific genetic forms of PD, and (3) enable the establishment of well‐defined subcohorts (ie, different genetically defined groups of PD) for further analyses and follow‐up studies. The ROPAD study enrolls participants who have been diagnosed with PD and individuals who are at risk of manifesting a genetic form of the disease. Within an anticipated 30‐month recruitment period, approximately 100 study centers in more than 15 countries will be established. The planned total number of study participants is 10,000.

The ROPAD study has been approved by the Ethics Committee (EC) at the Medical Faculty of the University of Rostock (A 2019–0017), central and local institutional review boards, and central and local ECs of participating sites (listed in Supporting Information Methods S1). ROPAD is registered at www.clinicaltrials.gov (Identifier: NCT03866603). It is part of a larger collaboration between CENTOGENE GmbH, the University of Lübeck (Lübeck, Germany), and Denali Therapeutics Inc. (San Francisco, CA).

### Inclusion Criteria

Recruited participants have to meet at least 1 of the following criteria: (1) clinical diagnosis of PD (cohort A), (2) first‐ or second‐degree relative of a participant who is positive for a pathogenic or likely pathogenic *LRRK2* variant (cohort B), (3) North African Berber or Ashkenazi Jew (cohort C). For qualifying individuals who are at least 18 years old and consent to participation, a neurological examination is performed, medical and family histories are documented, and a dried blood spot (DBS) sample is collected.

Individuals with positive genetic findings are offered enrollment in the LIPAD study. This observational study, performed at the University of Lübeck (Germany), aims at comprehensive clinical examination of participants in a longitudinal follow‐up setting. In addition, ROPAD participants harboring pathogenic *LRRK2* variants or other PD risk factors might be offered enrollment in future clinical trials with Denali Therapeutics Inc. and with other clinical trials.

### Genetic Screening

**FIG. 1:**
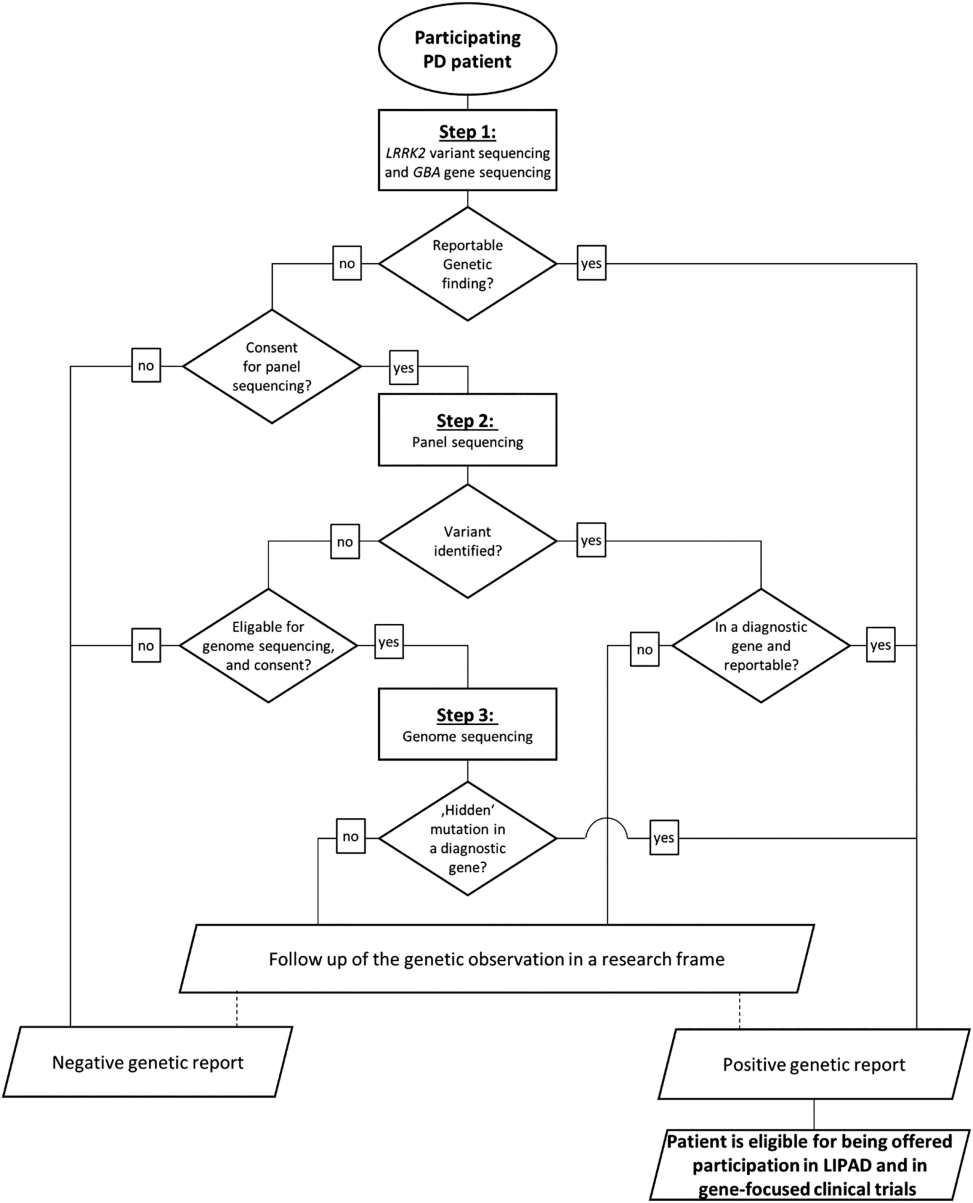
Workflow for genetic analysis of participants in cohort A.

Genomic DNA from DBSs is extracted and analyzed at CENTOGENE GmbH in a 3‐step screening approach for cohort A (Fig. 1). Step 1, performed for all participants, tests the presence of 11 pathogenic or likely pathogenic *LRRK2* variants (Supporting Information Table S1) and analyzes the *GBA* coding sequence by an approach that combines a primary *GBA*‐specific long‐range polymerse chain reaction (PCR) with subsequent *GBA* exon‐specific PCR and next‐generation sequencing of the resulting products.^11^ Given that variants in these 2 genes comprise the most frequent causes of monogenic PD, step 1 contributes considerably to an overall cost‐efficiency. If no reportable *LRRK2* or *GBA* variants are identified (see Supporting Information Methods S1 for reporting policy) and a subject has consented for further analyses, a next‐generation sequencing panel targeting 68 genes that have an established or suspected relevance for PD is applied as step 2 (Supporting Information Table S2). In addition, the gene panel‐derived data are analyzed for copy number variants (CNVs). Negative findings in the first 2 steps, combined with strong evidence for a genetic etiology (defined as positive PD family history or disease onset at before age 56 years), triggers genome sequencing if consented on enrollment. For cohorts B and C, screening step 1 is performed only. Further details regarding data management, reporting of genetic findings, and statistical analyses can be found in Supporting Information Methods S1.

## Results

### Initial Recruitment Data

Eight months into the ROPAD study, a total of 1360 participants have been enrolled. Whereas 67 individuals belonged to cohorts B or C and 5 patients did not consent for the PD panel, 1288 participants from cohort A were eligible for comprehensive testing until step 2 of the study workflow (Fig. 1). The following description focuses on the latter, that is, on unrelated participants who have received a clinical diagnosis of PD and consented for the panel testing (Table 1).

**TABLE 1:** Key categorical (A) and numerical (B) parameters of the cohort of 1288 ROPAD participants covered by the present study

A		
Parameter	Category	Number of patients (% of total)
Sex	Male	793 (61.6%)
	Female	495 (38.4%)
Race	Whites	1232 (96.4%)
	Hispanics	22 (1.7%)
	Asians	11 (0.9%)
	Black Africans	7 (0.5%)
	Others	6 (0.5%)
Family history	Positive	353 (27.4%)
	Negative	917 (71.2%)
	Not provided	18 (1.4%)
On l‐dopa treatment at inclusion	Yes	1126 (87.4%)
	No	155 (12.0%)
	Not provided	7 (0.5%)

B		
Parameter	Mean ± SD, y	Median (range), y
Age at disease onset	60.2 (11.5)	61 (16–92)
Age at enrollment	67.0 (10.7)	68 (19–92)

The 1288 patients were recruited by 29 centers (1–295 patients per center; median, 20) from 8 countries (7–492 patients per country; median, 51.5). There were 793 male participants (61.6%), and whites represented the major ethnicity (n = 1232; 95.7%). Age at onset (mean ± SD, 60.2 ± 11.5 years; median, 61 years; range, 16–92 years) preceded age at enrollment (mean ± SD, 67.0 ± 10.4 years; median, 68 years; range, 19–92 years) by approximately 7 years. Family history of PD was positive for 353 patients (27.4%). At the time of inclusion, 1126 patients (87.4%) were receiving l‐dopa.

### Initial Genetic Findings

Step 1 of the ROPAD genetic analysis pipeline was successfully accomplished in all 1288 PD patients, identifying 152 participants with positive genetic findings. Of those, 40 (3.1% of all) harbored pathogenic or likely pathogenic *LRRK2* variants, 109 (8.5%) harbored reportable *GBA* variants, and 3 (0.2%) had alterations in both genes. The remaining 1136 patients (88.2%) entered step 2. PD panel sequencing has been completed in 807 of those participants, with reportable variants detected in 17 individuals (2.1%). The majority of these cases carried homozygous or (potentially) compound‐heterozygous variants in *PRKN* (n = 10, 58.8%), whereas 7 participants received 7 other distinct genetic diagnoses. The first 2 steps of our study have thus resulted in the identification of 169 patients with a genetic etiology of PD. Extrapolating from the 11.8% and 2.1% positive findings in steps 1 and 2, respectively, the current data correspond to a genetic diagnostic yield of ~14%.

The most frequently detected *LRRK2* variant was c.6055G>A (p.Gly2019Ser), found in 23 cases. In *GBA*, the 2 most prevalent variants were c.1093G>A (p.Glu365Lys) and c.1223C>T (p.Thr408Met), identified in 35 and 32 participants, respectively. In *PRKN*, the third most frequently affected gene, 8 distinct CNVs accounted for 11 of the 20 mutant alleles. A heterozygous duplication of the whole *SNCA* gene was another remarkable finding.

### Initial Genotype‐Associated Observations

In participants with negative findings in steps 1 and 2 (n = 790), the mean age at onset (AAO) was 58.5 ± 11.3 years, and thus comparable to the mean AAO of *LRRK2* variant carriers (59.4 ± 11.5 years; *P* = 0.59). In contrast, the mean AAO in *GBA* variant carriers (55.8 ± 10.0 years) and in patients with *PRKN*‐associated PD (43.5 ± 17.8 years) was significantly lower (*P* = 0.02 and *P* < 0.01, respectively). The male‐to‐female ratio was 1.68 in the patients with no detected PD‐related variants. This proportion was not significantly different in those *GBA* risk‐factor positive (1.33; *P* = 0.30). However, the individuals with either genetically confirmed *LRRK2*‐ or *PRKN*‐associated PD displayed a trend toward a lower male‐to‐female ratio (1.00; *P* = 0.06).

## Discussion

Carefully designed and executed epidemiological studies are a prerequisite for urgently needed translational and interventional PD research. In line with this, the primary objective of the ROPAD study is to determine the prevalence of different genetic PD forms among ~10,000 study participants. Currently, PD patients represent nearly 95% (1288 of 1360) of enrolled participants. Even at this very early stage of ROPAD, they comprise the largest PD cohort systematically and comprehensively screened for all known monogenic disease causes to date. Based on the extrapolation of the initial data, we estimate that up to 1400 of the ROPAD‐enrolled PD patients will receive a positive genetic test report. In nonpatient cohorts B and C, we expect to identify a considerable number of additional individuals who are positive for the *LRRK2* or *GBA* variant. These will be of high value for follow‐up studies focusing on, for example, penetrance or the development of biomarkers for the corresponding genetic forms.

Thus far, our enrollment strategy has yielded a typical PD cohort in terms of AAO, sex distribution, and family history.^12^, ^13^, ^14^ Furthermore, the frequencies of variants in *LRRK2*, *GBA*, and *PRKN* are comparable to findings from previous screening studies targeting these 3 genes in single cohorts.^3^, ^15^, ^16^ As in the aforementioned reports, p.Gly2019Ser is the most frequent *LRRK2* variant and p.Glu365Lys and p.Thr408Met are the most prevalent *GBA* alleles.^15^, ^16^ Notably, we have confirmed the observation that some patients might harbor variants in both *LRRK2* and *GBA*.^15^ The *PRKN* and *SNCA* CNVs in our patient cohort highlight the importance of a CNV pipeline, which is often not included in the analysis of gene panel sequencing data in other studies. As expected, *GBA* and *PRKN* variants were associated with lower AAO in our preliminary patient cohort,^17^, ^18^ whereas AAOs of patients with *LRRK2* alterations were comparable to those without monogenic PD cause.^19^ Finally, the proportion of male and female participants was approximately equal in individuals with *LRRK2* and *PRKN* changes, whereas the male‐to‐female ratio was higher among *GBA* variant carriers and patients without PD‐relevant alterations, as previously reported.^18^, ^19^, ^20^

The individuals with positive genetic findings will be offered participation in the LIPAD study, which may contribute to uncovering valuable novel insights into PD. In combination, ROPAD and LIPAD can be expected to shed further light on numerous aspects that are of crucial value for the development of therapeutic strategies in PD, including reduced penetrance^21^, ^22^, ^23^ and variable expressivity.^24^, ^25^ Importantly, the ROPAD participants receiving a genetic diagnosis may be offered participation in ongoing or planned gene‐tailored clinical trials. ROPAD and LIPAD will also facilitate the development of diagnostic and monitoring biomarkers in well‐defined PD subgroups, in contrast to the heterogeneous participant populations that are typically employed for such discovery studies.^26^ Finally, the genome sequencing performed as step 3 of our study may (1) disclose ‘hidden’ variants (deep intronic or regulatory alterations, balanced/complex rearrangements, etc.) in known genes, (2) confirm PD gene candidates as suggested in previous studies, and/or (3) lead to the identification of novel PD genes. Given that the majority of genetic PD forms resemble idiopathic PD at clinical, neuropathological, and pathophysiological levels;^9^, ^27^, ^28^, ^29^ further understanding of genetic PD through ROPAD may eventually also provide insights into various aspects of idiopathic PD.

### Appendix

**ROPAD study group investigators** (in alphabetical order): Jan Aasly, St. Olav's Hospital, Trondheim, Norway; Pinky Agarwal, Booth Gardner Parkinson's Care Center, Kirkland, WA; Jason Aldred, Inland Northwest Research, Spokane, WA; Roderick Anderson, Tucson Neuroscience Research, Tucson, AZ; Perminder Bhatia, Neuro‐Pain Medical Center, Fresno, CA; Ilona Csoti, Gertrudis‐Kliniken im Parkinson‐Zentrum, Leun‐Biskirchen, Germany; Paskal Cullufi, University Hospital Center “Mother Teresa”, Tirana, Albania; Aaron Ellenbogen, Michigan Institute for Neurological Disorders, Farmington Hills, MI; Sibel Ertan, Koç University School of Medicine, Istanbul, Turkey; Giorgio Fabiani, Hospital Angelina Caron, Curitiba, Brazil; Björn Falkenburger, Universitätsklinikum Carl Gustav Carus, Dresden, Germany; Gerald Ferencz, Neuroscience Research Institute of NJ, Toms River, NJ; Mark Gudesblatt, South Shore Neurologic Associates, New York; Stuart Isaacson, Parkinson's Disease and Movement Disorder Center of Boca Raton, Boca Raton, FL; Singar Jagadeesan, M3 Wake Research Inc., Raleigh, NC; Christine Klein, Universität Lübeck, Lübeck, Germany; Omesh Kulkarni, Royal Preston Hospital, Preston, U.K.; Bennett Myers, Dent Neurologic Institute, Amherst, MA; Anette Nieves, Renstar Medical Research, Ocala, FL; Nicola Pavese, Newcastle University, Newcastle upon Tyne, U.K.; Jason Raw, Fairfield General Hospital, Bury, U.K.; Burton Scott, Duke University, Durham, NC; Stuart Shafer, Vero Beach Neurology & Research Institute, Vero Beach, FL; Lars Tönges, St. Josef‐Hospital, Bochum, Germany; Peter Urban, Asklepios Klinik Barmbek, Hamburg, Germany; Enza Maria Valente, Università di Pavia, Pavia, Italy.

## Author Roles

1. Research project: A. Conception, B. Organization, C. Execution;
2. Statistical Analysis: A. Design, B. Execution, C. Review and Critique;
3. Manuscript Preparation: A. Writing of the first draft, B. Review and Critique.

V.S.: 1A, 1B, 1C, 2A, 2B, 3A, 3B; H.G.: 1B, 1C, 3B; E.‐J.V.: 1A, 1B, 3B; T.F.: 1B, 1C, 3A, 3B; T.U.: 1A, 1B, 3B; F.C.: 1B, 1C, 3B; N.B.: 1A, 1B, 3B; J.P.: 1B, 1C, 3B; X.B.: 1B, 1C, 3B; S.Z.: 1B, 1C, 3B; M.O.: 1B, 1C, 3B; S.S.: 1B, 1C, 3B; N.A.: 1B, 2A, 2B, 3B; P.B.: 1A, 1B, 1C, 2C, 3B; I.C.: 1B, 1C, 3B; N.K.‐A.: 1B, 1C, 3B; U.G.: 2A, 2B, 2C, 3B; A.W.: 2A, 2B, 3A, 3B; M.K.: 1A, 2A, 3B; C.B.: 1B, 2A, 2B, 3A, 3B; C.K.: 1A, 1B, 2A, 2C, 3B; A.R.: 1A, 1B, 2A, 2C, 3B.

## Supporting information


**Table S1.** List of *LRRK2* variants screened for in ROPAD's analytical step 1
**Table S2.** List of 68 genes screened by panel sequencing in ROPAD's analytical step 2 (diagnostic genes have strong support for a role in the etiology of PD, whereas research genes are hypothesized genetic factors)
**Table S3.** Content of electronic case report form
