# Supplementary material for: The Rostock International Parkinson's Disease (ROPAD) Study: Protocol and Initial Findings
Source: Mov Disord. 2020 Dec 14;36(4):1005–10. doi: 10.1002/mds.28416 (PMC8246975; doi:10.1002/mds.28416)
Supplement: Supplementary file 1 — Table S1. List of LRRK2 variants screened for in ROPAD's analytical step 1 Table S2. List of 68 genes screened by panel sequencing in ROPAD's analytical step 2 (diagnostic genes have strong support for a role in the etiology of PD, whereas research genes are hypothesized genetic factors) Table S3. Content of electronic case report form [file MDS-36-1005-s001.docx]

**SUPPORTING INFORMATION**

**Rostock International Parkinson’s Disease (ROPAD) Study: Protocol and Preliminary Data**

Volha Skrahina, PhD,^1^ Hanaa Gaber, PhD,^1^ Eva-Juliane Vollstedt, MD,^2^ Toni M. Förster, PhD,^1^ Tatiana Usnich,^2^ MD, Filipa Curado, PhD,^1^ Norbert Brüggemann,^2^ MD, Jefri Paul, PhD,^1^ Xenia Bogdanovic, PhD,^1^ Selen Zülbahar, PhD,^1^ Maria Olmedillas, PhD,^1^ Snezana Skobalj, PhD,^1^ Najim Ameziane, PhD,^1^ Peter Bauer, MD,^1^ Ilona Csoti, MD,^3^ Natalia Koleva-Alazeh, MD,^3^ Ulrike Grittner, PhD,^1^ Ana Westenberger, PhD,^1^ Meike Kasten, MD,^2^ Christian Beetz, PhD,^1,*^ Christine Klein, MD,^2^ Arndt Rolfs, MD,^1^ and the ROPAD Study Group^4^

^1^ CENTOGENE GmbH, Rostock, Germany

^2^ Institute of Neurogenetics, University of Lübeck, Lübeck, Germany

^3^ Parkinson-Center, Gertrudisklinik Biskirchen, Leun, Germany

^4^ investigators are listed as appendix

**Methods S1**

**Approval by institutional review boards**

The Rostock International Parkinson’s Disease (ROPAD) study has been approved by the by the following local and central Institutional Review Boards and Ethics Committees: EC TU Dresden: EK 346072019; EC LÄK Hessen: MC 99/2019; EC LÄK Hamburg: MC-150/19; EC Lübeck: 19-216; EC RU Bochum: 19-6751-BR; EC Istanbul: 2019.251.IRB1.048; Ministria e Shëndetësisë KOMITETI I ETIKES 147/22; Brazil CONEP 3.890.159; EC Italy Pavia P-20190077562; EC UK IRAS project ID: 264442, REC reference: 19/LO/0893; EC USA IRB Tracking Number: 20191177.

**Data management**

For each participant, a study-specific developed Case Report Form (CRF) is completed at the recruitment site. CRF covers demographic, clinical, and treatment data (Supporting Information Table S3). All participants’ data are pseudonymized and files containing participant IDs are secured with a password, solely known by study staff at the centers. The pseudonymized central electronic database is hosted and managed at CENTOGENE GmbH. High overall data quality is achieved by routine database checks; missing and/or implausible data trigger follow-up communication with the contributing centers.

**Reporting of genetic findings**

Out of 68 targeted within a next-generation sequencing panel applied as step 2, for 47 genes (‘diagnostic gene set’) an association with PD or with clinically overlapping disorders has been established, while the remaining 21 genes (‘research gene set’) were included based on a suggested, but currently not well-established association with PD (Supporting Information Table S2). Genetic variants in the ‘diagnostic gene set’ are classified according to the consensus recommendations of the American College of Medical Genetics and Genomics.^1^ Variants in *GBA* are considered risk factors for PD when they have been classified as pathogenic or likely pathogenic for GD (according to CentoLSD^TM^ [https://www.centogene.com/centolsd.html]), or when they have been observed as significantly enriched in PD patients vs. controls.^2^ A positive genetic diagnostic report is issued upon i) identification of pathogenic or likely pathogenic variants in the ‘diagnostic gene set’ in the relevant zygosity or ii) identification of a *GBA* risk factor.

**Statistical analyses**

The primary objective of the ROPAD study is to estimate the prevalence of patients with specific genetic findings among cohort A of our study population (compare inclusion criteria). This will be done by calculating an exact two-sided 95% confidence interval (CI) based on assuming a binomial distribution. A secondary objective is to identify correlations between the genetic observations and parameters such as age at onset, presence/absence of specific symptoms, disease severity, family history etc. Univariate descriptive statistics will include the presentation of means and standard deviation or median and interquartile ranges for continuous measures depending on the distribution of these measures, and absolute and relative frequencies for categorical variables. For the comparison of groups, we will present standardized mean differences and 95% CI as standardized effect estimates. Multiple regression models will be used to control for possible confounders. Regression models will additionally account for center heterogeneity by including a random intercept for each center.

**References for Supplemental Methods**

1. Richards S, Aziz N, Bale S, et al. Standards and guidelines for the interpretation of sequence variants: a joint consensus recommendation of the American College of Medical Genetics and Genomics and the Association for Molecular Pathology. Genet Med 2015; 17: 405–423.

2. Zhang Y, Shu L, Sun Q, et al. Integrated genetic analysis of racial differences of common GBA variants in Parkinson’s disease: A meta-analysis. Frontiers in Molecular Neuroscience; 11. Epub ahead of print February 15, 2018. DOI: 10.3389/fnmol.2018.00043.

**Table S1. List of *LRRK2* variants screened for in ROPAD’s analytical step 1.**

| **cDNA nomenclature** | **Protein nomenclature** |
| --- | --- |
| c.6055G>A | p.(Gly2019Ser) |
| c.4322G>A | p.(Arg1441His) |
| c.4321C>T | p.(Arg1441Cys) |
| c.4321C>G | p.(Arg1441Gly) |
| c.4522A>G | p.(Ser1508Gly) |
| c.4309A>C | p.(Asn1437His) |
| c.4541G>A | p.(Arg1514Gln) |
| c.6059T>C | p.(Ile2020Thr) |
| c.5096A>G | p.(Tyr1699Cys) |
| c.6035T>C | p.(Ile2012Thr) |
| c.4391C>G | p.(Ala1464Gly) |

**Table S2. List of 68 genes screened by panel sequencing in ROPAD’s analytical step 2. Diagnostic genes have strong support for a role in the etiology of PD, while research genes are hypothesized genetic factors.**

| diagnostic genes  (n=47) | *ADCY5; ANO3; APP; ATP13A2; ATP1A3; C19orf12; CHCHD2; COX20; DCTN1; DJ1; DNAJC13; DNAJC6; FBXO7; GBA; GCDH; GCH1; GNAL; GNE; HPCA; KCTD17; KMT2B; LRRK2; MAPT; PANK2; PARK2; PDE8B; PDGFB; PDGFRB; PINK1; PLA2G6; PRKRA; Rab39B; SGCE; SLC19A3; SLC20A2; SLC30A10; SLC39A14; SLC6A3; SNCA; SYNJ1; TAF1; THAP1; TOR1A; VAC14; VPS13C; VPS35; XPR1* |
| --- | --- |
| research genes  (n=21) | *APOE; ATP9A; CNEP1R1; CTDNEP1; ELOVL7; FBXO47; GAK; GRN; LPIN1; LPIN2; LPIN3; MCCC1; MCOLN1; NPC1; POLG; PSEN1; PSEN2; Rab12; SNCB; SYN1; TARTDP* |

**Table S3. Content of electronic Case Report Form.**

| Demographics | For all participants: age, gender at birth, race; for PD participants: age at disease onset, age at PD clinical diagnosis |
| --- | --- |
| Symptoms | Tremor, impaired smell, constipation, daytime somnolence, orthostatic hypotension, urinary dysfunction, cognitive deficits, impaired posture, gait difficulties |
| Comorbidities | Diabetes, arterial hypertension, atrial fibrillation / cardiac arrhythmias, peripheral artery disease, gastrointestinal disease, stroke/ transient ischemic attack/cerebral haemorrhage |
| PD criteria | Bradykinesia, resting tremor, rigidity, response to dopaminergic therapy, L-dopa-induced dyskinesia, olfactory loss |
| Medication | L-dopa, Entecapone/Tolcapone/Opicapone; monoamine oxidase (MAO)-Inhibitors: Moclobemide or Selegiline or Rasagiline; any other PD-specific drugs: Pramipexole, Ropinirole, Rotigotine, Piribedil, Safinamide, Bromocriptin, Pergolid, Cabergolin, Amantadine |
| Family history | Family relation to PD and tremor patients |
| Clinical score | Shortened MDS-UPDRS (Movement Disorder Society – Unified Parkinson’s Disease Rating Scale) part III |
